# Supplementary material for: Protocol for the systematic review of the Pneumocystis jirovecii-associated pneumonia in non-HIV immunocompromised patients
Source: PLoS One. 2024 May 9;19(5):e0302055. doi: 10.1371/journal.pone.0302055 (PMC11081338; doi:10.1371/journal.pone.0302055)
Supplement: Attachment — Submitted filename: Response to Reviewer letter MEOU Plos One.pdf [file pone.0302055.s006.pdf]

March 17, 2024

Benjamin M. Liu, MBBS, PhD.
PLOS ONE
Public Library of Science
1160 Battery Street, Suite 100
San Francisco, CA 94111
USA

Dear Dr. Benjamin M. Liu,

Thank you for your detailed comments on our manuscript titled *"Protocol for a Systematic Review on Pneumocystis jirovecii-associated Pneumonia in Non-HIV Immunocompromised Patients"*. We have carefully reviewed your suggestions and have also considered the reviewer's comments. Below, we present our responses to each point raised:

Line 91-103:

Response: We have updated the manuscript's introduction to include information on novel molecular testing for Pneumocystis jirovecii pneumonia (PCP). We have cited the provided reference and discussed the advances and challenges of PCR for PJP detection, including increased sensitivity, fast turnaround time, and challenges associated with differentiation between colocalization and true infection. Additionally, we have highlighted the work of Liu et al. (2020) and its impact in this field.

The number of paragraphs in the Discussion section:

Response: We have revised the manuscript's structure to ensure a broader and more detailed discussion of different aspects of PJP in the Discussion section. Our protocol follows the guidelines for PLOS ONE authors, emphasizing the strengths and limitations of the protocol, as well as the potential implications of the review. The Discussion section now encompasses multiple paragraphs exploring various aspects related to PJP, including clinical aspects, diagnosis, treatment, and future perspectives.

Line 234 - Reviewer's Comment:

We confirmed that this systematic review will utilize published data exclusively and does not involve any human subjects or data collection from individuals. Therefore, it does not necessitate ethical approval from the ethics committee of our faculty or university. We have clarified this point in the revised version of the manuscript.

We have attached the revised version of the manuscript with all modifications highlighted for your review. We are confident that these updates will significantly enhance the quality and scope of our work for the PLOS ONE audience.

We remain available for any further clarification or information required. We appreciate once again the opportunity to improve our manuscript and look forward to the possibility of its consideration for publication in PLOS ONE.

Mauricio E. Orozco-Ugarriza
Universidad de San Buenaventura, Cartagena, Colombia.
E-mail: mauricioorozcou@gmail.com, morozcou@usbcartagena.edu.co.
